# Predictions from algorithmic modeling result in better decisions than from data modeling for soybean iron deficiency chlorosis

**DOI:** 10.1371/journal.pone.0240948

**Published:** 2021-07-09

**Authors:** Zhanyou Xu, Andreomar Kurek, Steven B. Cannon, William D. Beavis

**Affiliations:** 1 Plant Science Research Unit, USDA, Agricultural Research Service, Saint Paul, MN, United States of America; 2 Department of Agronomy, Iowa State University, Ames, IA, United States of America; 3 Corn Insects, and Crop Genetics Research Unit, USDA, Agricultural Research Service, Ames, IA, United States of America; Julius Kuhn-Institut, GERMANY

## Abstract

In soybean variety development and genetic improvement projects, iron deficiency chlorosis (IDC) is visually assessed as an ordinal response variable. Linear Mixed Models for Genomic Prediction (GP) have been developed, compared, and used to select continuous plant traits such as yield, height, and maturity, but can be inappropriate for ordinal traits. Generalized Linear Mixed Models have been developed for GP of ordinal response variables. However, neither approach addresses the most important questions for cultivar development and genetic improvement: How frequently are the ‘wrong’ genotypes retained, and how often are the ‘correct’ genotypes discarded? The research objective reported herein was to compare outcomes from four data modeling and six algorithmic modeling GP methods applied to IDC using decision metrics appropriate for variety development and genetic improvement projects. Appropriate metrics for decision making consist of specificity, sensitivity, precision, decision accuracy, and area under the receiver operating characteristic curve. Data modeling methods for GP included ridge regression, logistic regression, penalized logistic regression, and Bayesian generalized linear regression. Algorithmic modeling methods include Random Forest, Gradient Boosting Machine, Support Vector Machine, K-Nearest Neighbors, Naïve Bayes, and Artificial Neural Network. We found that a Support Vector Machine model provided the most specific decisions of correctly discarding IDC susceptible genotypes, while a Random Forest model resulted in the best decisions of retaining IDC tolerant genotypes, as well as the best outcomes when considering all decision metrics. Overall, the predictions from algorithmic modeling result in better decisions than from data modeling methods applied to soybean IDC.

## Introduction

**Fig 1:**
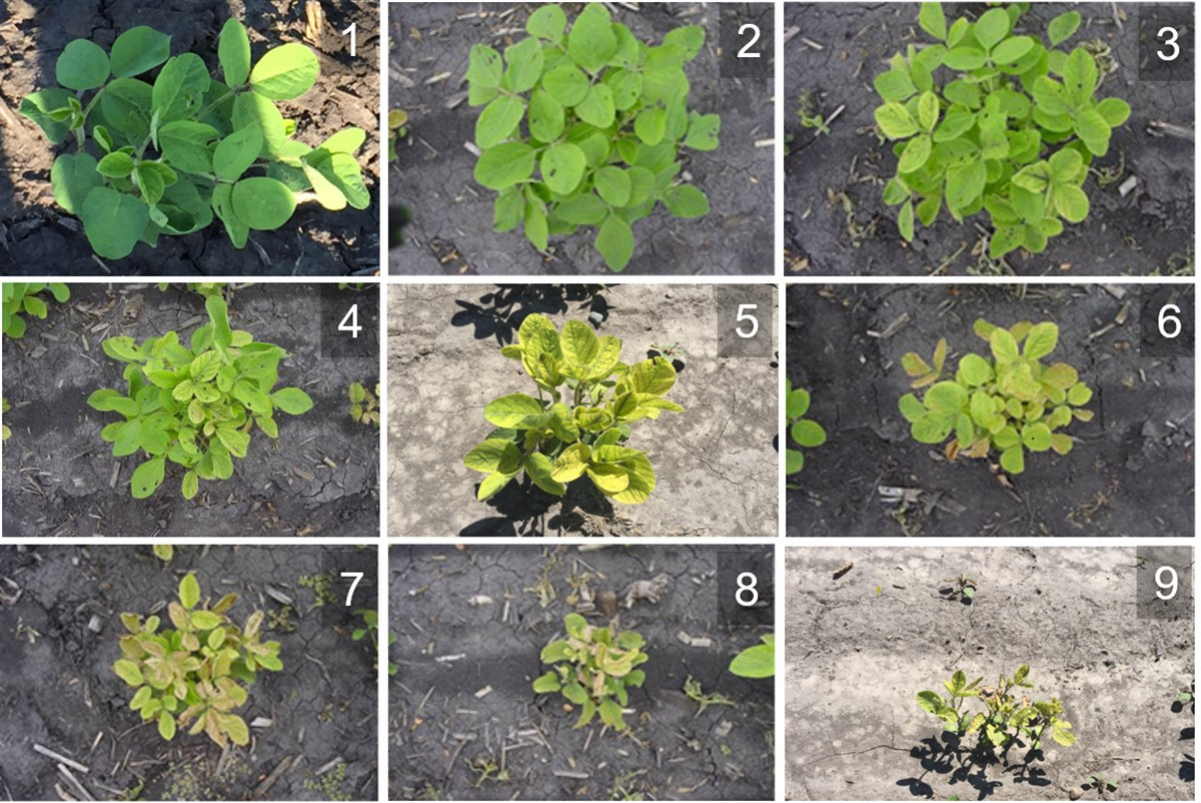
Soybean IDC ordinal scores on a 1 to 9 scale, where an observed score of 1 is applied to plots that are completely tolerant to IDC conditions, and 9 is applied to plots with plants that are highly susceptible to IDC conditions.

Iron deficiency chlorosis (IDC) in soybean is associated with yield losses of 340 million tons, worth an estimated $120 million per year [1]. However, breeding for IDC tolerance in soybean is time-consuming and expensive. Soybean variety development, like cultivar development projects in most commodity crops, can be represented as a pipeline consisting of a series of development and evaluation stages. In all crops, the first stage is to make dozens to hundreds of crosses [2]. After crossing, only time and a little labor are needed to develop replicable, i.e., homozygous genotypes, also known as lines [3]. After lines have been developed, they are evaluated in a preliminary yield trial (PYT), usually consisting of tens of thousands of lines with each line evaluated in a small number of replicated field plots. Depending on the number of available field plots budgeted for subsequent field trials, a proportion of the lines that exhibit low yields in the preliminary field trial will not be retained for evaluation in additional annual stages of field trials. After the PYT, soybean variety development in the upper Midwest (Iowa, Minnesota, and North Dakota) includes large-scale screening experiments to evaluate lines in soils that have high levels of carbonates and/or soluble salts resulting in expression of IDC (Fig 1). Because expression of IDC depends on ephemeral environmental conditions [4], obtaining repeatable IDC scores can require multiple attempts with many combinations of years and locations. Thus, IDC was identified as a trait that will benefit from the application of marker-assisted selection (MAS).

Initially, the effort to develop MAS methods for IDC was based on finding statistical associations between segregating molecular markers and IDC scores (QTL). After the first QTL were identified for IDC, commercial soybean breeders attempted to use marker alleles to stack desirable IDC QTL alleles but without successful outcomes. In contrast to many disease resistance QTL studies in soybean, there have been few consistently identified QTLs with large estimated genetic effects for tolerance to IDC. The exception was a single large-effect IDC QTL that explained more than 70% of the phenotypic variation in a sample of lines derived from Anoka x A7 [5, 6]. However, no reports have found that this IDC QTL provides resistance in other genetic backgrounds. Indeed, identified IDC QTL appear to be highly dependent on genetic background [7]. SoyBase [8] indicates that 41 IDC QTLs have been identified using bi-parental linkage studies, 50 QTLs have been identified using connected network studies, and 88 QTLs have been identified using Genome Wide Association Studies (GWAS) [4, 7, 9–14]. In addition, 835 candidate genes in the IDC resistant line Clark (PI548553) were identified by transcriptome sequencing [15]. To summarize, tolerance to IDC is a highly desired agronomic trait with economic benefits to farmers, but it is also a complex trait that exhibits transient expression. Thus, it is a trait that would benefit from MAS, but QTL-based MAS for IDC tolerance has been ineffective [16, 17].

Genomic selection (GS) was developed as a high-density marker-based method to obtain genomic estimated breeding values (GEBV’s) for purposes of improving complex polygenic traits [18]. The efficiency and effectiveness of GS depend, in part, on the genomic prediction (GP) model that utilizes both phenotypic data and genotypic data from high-density genotyping or whole-genome sequencing technologies [18, 19]. Results from applications of GS to quantitative traits indicate that it provides greater responses to selection than linkage-based selection for QTL in both empirical and simulation experiments [20–24]. About 20 GP methods have been proposed, developed, and compared [23–25]. These GP models can be classified as belonging to two groups, described by Breiman as "data modeling" and "algorithmic modeling" approaches [26].

The data modeling approach uses linear or non-linear models composed of fixed and random effect parameters. Most proposed GP methods belong to the data modeling approach based on the Linear Mixed Model (LMM) framework, which assumes that the traits of interest are continuous, with normal, identically distributed random effects. A best linear unbiased prediction algorithm using ridge-regression (RR) is the most widely used LMM method for GP, largely because it has been available as the R package rrBLUP [27, 28]. However, the underlying assumptions for obtaining genomic BLUP values with RR are violated for many economically important traits, such as IDC [29, 30]. Stroup [31] demonstrated that violating the assumptions about the distributions of the response variable and the random effect parameters of the model’s independent variables can result in poor decisions. He also demonstrated how these poor decisions can be corrected using the Generalized Linear Mixed Model (GLMM) framework. The concept of assigning a non-Normal distribution to random effects in a model is easily accommodated with Bayesian prior distributions. For example, Bayesian Logistic Ordinal Regression (BLOR) methods were proposed for GP [32] and reviewed by Montesinos-López et al. (2017). These methods have been implemented in the BGLR R package [33].

In contrast to the data modeling methods, algorithmic modeling methods make no assumptions about the distribution of the response variables, linear or non-linear relationships among parameters in a model, nor about the distributions of random effect parameters. Examples of algorithmic models include Random Forest classification, Support Vector Machines, Artificial Neural Networks, and Deep Learning algorithms. Algorithmic models are typically applied to large complex data sets and have become known by plant breeders as machine learning analysis tools.

Most comparative studies of GP methods have been conducted on continuous traits [22, 34–39]. The most often used criterion for comparing GP methods is prediction accuracy, calculated as the Pearson correlation coefficient (r_gm_) between "true" genotypic values g, and genotypic values predicted by marker effects, m. The true genotypic values are seldom known, except in simulation experiments or determined by validation using large numbers of replicated phenotypes. Alternatively, r_gm_ is calculated as r_pm_/h, the correlation coefficient between predicted phenotypic values, p, and marker predicted genotypic values, m, divided by the square root of the estimated heritability. Prediction accuracies of quantitative traits evaluated in field trials range from -0.59 to 1.0 depending on the heritability of the trait and assumed underlying genetic architectures [40]. However, in the context of variety development, comparisons among GP methods using r_gm_ do not address whether desired genotypes are correctly retained nor if undesirable genotypes are correctly discarded. Plant breeding is a decision-making discipline. In the context of variety development, decisions are binary: advance to the next stage or don’t advance to the next stage. Thus, in addition to estimates of r_gm_, decision metrics such as sensitivity, specificity, precision, decision accuracy, and the area under the receiver operating characteristic curve (defined in the Methods section below) are metrics that should be used when comparing results from analytic methods for cultivar development and genetic improvement.

Herein, we report a comparison of GP models using five metrics of relevance for plant breeding decisions in variety development and genetic improvement projects. Four methods consisting of ridge regression (RR), Bayesian Generalized Linear Regression(BGLR), Logistic regression(LR), and Penalized Logistic Regression(PLR) represent data modeling approaches. Six methods consisting of Naïve Bayes (NB), Random Forest (RF), K-nearest Neighbour (KNN), Support Vector Machine (SVM), Gradient Boost Machine (GBM), and Artificial Neural Network (ANN) represent algorithmic modeling approaches.

## Materials and methods

### Data

We obtained observed IDC ordinal scores from large-scale field screening trials conducted by a commercial soybean breeding program. Depending on the prevalence of ephemeral IDC conditions, high quality IDC scores might be obtained for a few hundred to many thousands of lines at each IDC environment (combination of location and year). The observed IDC scores were obtained using visual evaluations (Fig 1) of 38,803 experimental lines grown in 144,393 two-row field plots distributed among incomplete blocks in 48 environments located in North Dakota, Minnesota, and Iowa from 2013 to 2016. Experimental lines were grouped by families that were randomly assigned to IDC evaluation environments each year. Because the number of experimental lines per environment is large and IDC conditions are ephemeral and not homogeneous across the environment, plots at each IDC environment were blocked into smaller units, usually consisting of 42 plots, but occasionally as many as 84 plots. Experimental lines from the same family along with tolerant and susceptible "check" varieties augmented the experimental lines assigned to each incomplete block [41]. Reflecting the prevalence of IDC conditions, the number of blocks with useful IDC data per year ranged from 2,109 to 7,651, and the number of blocks per environment with informative IDC data ranged from six to well over 1,500.

Subsets of experimental genotypes and check varieties were randomly assigned to plots within each block. Since families of lines assigned to incomplete blocks were not of the same maturity, the check varieties assigned to blocks consisting of early maturing families were not the same as checks assigned to blocks consisting of late maturing families. However, since all maturity groups overlap, the check varieties between blocks of adjacent maturity groups also overlap. Thus the replicated check varieties are organized in a randomized connected block design. The IDC scores for 640 check varieties were distributed among ~18,000 blocks nested within 48 environments. Since the replicated check varieties provide information about consistency of IDC conditions among incomplete blocks within and among environments, the IDC values for the checks were used to obtain BLUP values of block effects (S8 File). These BLUP values for block effects were used as a covariate in the assessment of the proportion of phenotypic variability due to genotypic variability, denoted *i*^2^ [42], of IDC scores for experimental lines.

Across the years, these same experimental lines were evaluated for yield, height, and maturity at locations without high carbonates and/or soluble salts. Field trials for these other agronomic traits resulted in the retention of 1000 of the experimental lines. It is important to emphasize that selection between 2013 and 2016 was not based on IDC information. The subset of 1000 experimental lines were evaluated for IDC in 4,171 two-row field plots at 40 of the 48 environments from 2013 to 2016 (S9 File). The column labeled "obs_IDC_score" in S9 File consists of these field observed scores and was used for phenotypic analyses. In particular, an LMM was applied to the “obs_IDC_score” to estimate reliability [42] (*i*^2^ = σ^2^_g_/σ^2^_p_) on an entry mean basis and obtain an IDC BLUP value for each line (see below). Additional IDC field evaluations for these 1000 lines were used by a group of commercial soybean breeders to determine and validate a “true decision” about the tolerance of the line to IDC conditions. The 1000 IDC_BLUP values and true_decision outcomes were merged with genotypic data for the same set of lines (S10 File). The genotypic data were obtained with a 3K SNP Illumina chip and consisted of 1200 polymorphic SNP markers with minor allele frequencies greater than or equal to 0.05.

### Phenotypic analyses

Because environmental conditions were inconsistent among and within environments (year by location combinations) and since sets of check varieties assigned to each block overlapped but were not identical among all blocks, best linear unbiased predicted values for block effects were obtained using an LMM:

IDC=Cκ+Bπ+ε (1)

$$\pi ∼N(0,\;I\sigma^{2}{}_{blk}),$$

$$\varepsilon ∼N(0,\;I\sigma^{2}{}_{res}).$$

IDC represents a vector of 20,606 IDC scores for 640 check varieties evaluated in 3499 blocks in 48 environments (S8 File), **C** represents an incidence matrix for the check varieties, **κ** is the vector of unknown fixed effects represented by the check varieties, **B** is an incidence matrix indicating whether the IDC value was obtained from a block, **π** is the vector of unknown random effects represented by each block, **I** is the identity matrix, σ^2^_blk_ is the variance among blocks, **ε** represents residual values not accounted for in the model, and σ^2^_res_ is the variance among residual values. BLUP values for block effects were obtained using the lme4 package in R [42, 43].

Because there were variable numbers of experimental lines per environment and variable numbers of blocks within environments, an LMM was used to obtain IDC BLUP values for the 1000 experimental lines:

$$IDC=X\beta_{blk}+Z\upsilon +\varepsilon ,$$ (2)

$$\upsilon ∼N(0,\;I\sigma^{2}{}_{l}),$$

$$\varepsilon ∼N(0;\;I\sigma^{2}{}_{res}),$$

where **IDC** is a vector of 4,171 observed IDC scores for 1000 experimental lines evaluated in 40 of 48 environments (S9 File). **X** is an incidence matrix indicating whether the experimental line was evaluated in the block, β is a vector of predicted block effects and represents a fixed effect covariate, **Z** is an incidence matrix for experimental lines and **υ** is the vector of random effects for lines, **I** is the identity matrix, σ^2^_l_ is the genotypic variance among lines, **ε** represents the residual value, not accounted for in the model, and σ^2^_res_ is the variance among residual values.

Variance components were estimated using the lme4 R package [43]. Because the relationships among the experimental lines are unknown and there are unequal numbers of observations for each line, the estimated genotypic variance, σ^2^_g_, estimated residual variance σ^2^_**ε**_, and the harmonic mean η per experimental line [44], were used to estimate reliability as

$$\sigma^{2}{}_{g}/\sigma^{2}{}_{g}+\sigma^{2}{}_{\varepsilon }/\eta ),$$

usually incorrectly referred to as broad sense heritability [42]. The R code used to calculate reliability can be found in the S1 File.

### Model training and validation

For all GP methods, genotypic and phenotypic data in S10 File were randomly divided into 10 subgroups consisting of 100 lines per subgroup. Nine subgroups with 900 lines were used to train the model, and the remaining subgroup consisting of 100 lines was used to cross-validate the GBLUP genotypic values of the lines from the training set. Each of the 10 subgroups was used in turn to evaluate the stability of the model and whether the model is overfitted [45]. Ten-fold cross-validations for each model were repeated ten times to evaluate model stability.

### Genomic prediction methods

#### LMM: Genomic prediction with a continuous normally distributed data model

Assuming that IDC scores are distributed as continuous normal variables, the RR-BLUP method was used to obtain effects of genome-wide markers, which were subsequently used to obtain GBLUP genotypic values for the experimental lines:

IDC=1μ+Zm+ε, (3)

$$m∼N(0,\;I\sigma^{2}{}_{m}),$$

$$\varepsilon ∼N(0,\;I\sigma^{2}{}_{res}).$$

**IDC** is a 900 x 1 vector of IDC_BLUP values from (2) used in the training sets (S10 File), **1** is a 900x1 vector of 1’s, **μ** is the overall mean for the IDC_BLUP values (~4.33), **Z** is a 900x1200 matrix consisting of *z*_ij_ elements indicating whether the alleles at the j^th^ SNP locus for the i^th^ line is homozygous for a reference genotype (= 1), heterozygous (= 0) or homozygous for an allele that is not from the reference genotype (= -1). The vector of unknown random effects that need to be predicted from the 1200 SNP markers is designated as **m**, **I** is a 900 x 900 identity matrix, σ^2^_m_ is the genotypic variance for the markers, **ε** represents the 900 x 1 vector of residual values, and σ^2^_res_ is the residual variance. Note that with high densities of molecular markers, absence of major QTL effects, and QTL distributed uniformly across the genome, Habier et al [46] showed that the RR-BLUP method is equivalent to the GBLUP method originally proposed by Bernardo in 1994. The R package "rrBLUP" [27] was used to implement Eq (2) for the lines in each of the 10-fold cross validations (S2 File). The resulting GBLUP genotypic values ≥ 4 were associated with decisions to discard the line, and GBLUP genotypic values < 4 were associated with decisions to retain the line. Decisions based on GBLUP genotypic values produced using RR-BLUP were compared with “true_decisions” using five decision metrics (described below).

#### GLMM: Genomic prediction with Bayesian Generalized Linear Regression (BGLR)

The observed IDC scores are not continuous, rather they are values from a multinomial variable in which the process responsible for creating the observed categorical scores arises by applying a threshold model (also known as a cumulative probit model) to an underlying continuous normal variable (Fig 1). The resulting linear predictor model has the form:

$$\eta_{c}=\gamma_{c}‐X\beta ‐Z\upsilon ,$$

**η**_c_ is the link for the c^th^ category, γ_c_ is the threshold of the c^th^ link for the combinations of fixed, β, and random, **υ**, effects while **X** and **Z** are the incidence matrices for fixed and random effects. For our purposes, rather than utilizing BLUP values of block effects to represent variability among environments as a continuous fixed effect, we modeled environments as categorical fixed effects [47]. The elements of **η**_c_, denoted η_cge_, are the links for the c^th^ category of the g^th^ genotype (line) evaluated in the e^th^ environment. The cumulative probit was used as the link function for 900 genotypes replicated k times in e environments of the training sets (S9 File). Because we assume that the categories are derived from a threshold model, the underlying latent variable is modeled as a continuous variable with a normal distribution [32]:

$$l_{gek}=x^{T}{}_{ge}\beta +z^{T}{}_{ge}\upsilon +\varepsilon_{gek}$$ (4)

$$\varepsilon_{gek}∼N(0,1),\;thus$$

$$l_{gek}|\beta ,\upsilon ∼N(x^{T}{}_{ge}\beta +z^{T}{}_{ge}\upsilon ,\;1)$$

Based on prior work by Montesinos-López et al [32] we chose to model the latent variables with environments and lines modeled as fixed effects and random effects consisting of the marker derived genomic relationship matrix, additive x additive epistatic relationships, genotype x environment interaction effects, and epistatic x environment interaction effects. Montesinos-López et al [32] demonstrated that this model explained the greatest amount of variability among observed ordinal scores for gray leaf spot while at the same time providing among the best posterior predictions of breeding values for 278 maize lines. The R package BGLR [33] was used to conduct the analyses for each of the 10 fold cross-validations (S3 File). We summed the posterior probabilities for each category multiplied by IDC category values (1,2,3…9) to obtain Expected GBLUP (E-GBLUP) genotypic values for each experimental line. The resulting E-GBLUP genotypic values ≥ 4 were associated with decisions to discard the line and E-GBLUP genotypic values < 4 were associated with decisions to retain the line. Decisions based on E-GBLUP genotypic values produced using BGLR were compared with “true_decisions” using five decision metrics (described below).

#### GLMM: Genomic prediction using Logistic Regression (LR) and a Logit model

Because true decisions about IDC tolerance for the lines exist, this information, instead of the observed ordinal IDC scores, were used to develop a genomic prediction model. The binary response variable with two possible values can be viewed as a special case of the threshold model (4). The probability (π) of the true_IDC response variable = 1 can vary between 0 and 1 and the probability that line i = 1, (π_i_ = 1, i = 1,2,3…1000) is distributed as a binomial random variable. As a consequence, the logit link function η_i_ = logit (π_i_) = log(π_i_ /(1-π_i_)) [48] is applied to the response variable and a logistic regression model **η = Xβ + ε**, is built using a stepwise regression algorithm with significant genetic markers trait associations serving the role of explanatory variables. Estimated genetic effects of significant marker trait associations were used to obtain GBLUP decision values that were compared with “true_decisions” using five decision metrics (described below). An LR model was built with the R base package "stats," where the maximal number of reweighted least squares iterations was set to 50. The R code can be found in "S4 File".

#### GLMM: Genomic prediction by applying Penalized Logistic Regression (PLR) to the Logit model

Since the number of markers is greater than the number of lines, an alternative to LR is penalized logistic regression for binary traits: **η = 1μ** + **Zm** + **ε.** As with the RR-BLUP method, the ridge regression method of PLR shrinks the estimated marker effects [49], which are subsequently used to obtain GBLUP decision values for the experimental lines. The resulting GBLUP decision values were compared with “true_decisions” using five decision metrics (described below). The PLR method was implemented using the R package "glmnet" [50]. The R code can be found in "S5 File".

### Algorithmic modeling methods

Unlike data modeling methods, there are no assumptions about the distribution of response variables, nor are there any specified parameters with assumed distributions. Rather the predictor models are better represented as black box models:

K:m→A(m)→P(m),

where **K** represents a vector of known values for the response variable, **m** represents the matrix of genetic marker genotypic values, **A** represents the algorithm that finds patterns in **m** that are associated with **K** to produce predicted values **P** based on the patterns of **m**. Herein we use **K** consisting of 1000 true_decision outcomes (S10 File) and six versions of **A(m)**.

#### Artificial Neural Network (ANNs)

In the context of GP for decision making, ANNs model the relationship between a set of marker scores and true_decision scores that is analogous to an understanding of how a biological brain responds to stimuli from sensory inputs. ANN can be applied to solve complex but unknown relationships between variables and non-linear relationship between predictive and response variables [51]. ANN models "learn" from existing data and do not require a predefined model or statistical distribution [52, 53]. For modeling the relationship between the input and the ANN output, different neurons, so-called "hidden layers," are inserted by the computational algorithm between the predictive and response variables. These hidden layers are the ANN training processes that transform the input markers into a local output [54]. The ANN algorithms optimize the neurons’ weights in the hidden layers with regard to a task-specific prediction function [55]. Herein, the ANN model used two hidden layers with the backdrop algorithm to predict decisions via R package "neuralnet" [56]. The learning rate parameter was set to 0.01, and the maximum number of steps for training the neural network was set to 1,000,000. The ANN model and parameters for this study can be found in the supplemental R code ("S4 File")

*Random Forest* (RF) is an ensemble learning method for classification, regression, and feature selection that operates by computationally constructing multiple decision trees based on the set of molecular marker scores that could result in the IDC true_decision outcomes using a training set. After constructing the trees, the RF method will determine the mode of the classes (classification) or mean prediction among all possible decision trees. Random forests can handle large datasets, where the so-called "curse of dimensionality" might cause other models to fail. RF can extract important features and their patterns associated with the trait of interest with high prediction accuracy [26] and tends to be less prone to overfitting than bagging decision trees by pruning large trees [57]. Random forest classification was conducted with R package "randomForest". The number of trees selected for the analysis is 1000. The scripts can be found in the supplemental R markdown in "S4 File".

*Gradient Boosting Machine* (GBM) is a technique for classification based on decision trees. It allows iterative boosts to the performance of weak predictors to attain the performance of stronger predictors to improve the overall prediction accuracy [58]. GBM has recently dominated applied machine learning contests and has won most Kaggle competitions for structured or tabular data [59, 60]. GBM was conducted via R package XGBoost [61] with maximum step 10. The R code can be found from "S4 File".

*Support Vector Machine*s (SVM) are based on the concept of creating a computational boundary between multi-dimensional points of data and their feature values [62]. In the context of GP, the points of data represent molecular marker scores and the feature values represent IDC true_decision scores. The goal of an SVM algorithm is to create a flat boundary called a hyperplane, which divides the space to develop relatively homogeneous partitions on either side [63]. The SVM consists of a subset of data instances, so-called support vectors. They define a hyperplane separating classes into the feature space while its positions are unambiguously defined by the principle of maximum margin, thus facilitating the learning of models [55]. SVM analyses were conducted via R package "e1071" using linear kernel and cost = 01. The R code can be found in "S4 File".

*Naive Bayes* (NB) methods consist of a family of simple probabilistic classifiers based on applying Bayes’ theorem with strong independence assumptions among the features. NB Classifiers using naïve Bayes methods utilize training data to calculate an observed probability of each outcome based on the evidence provided by feature values [63]. NB is best applied to problems in which the information from numerous attributes should be considered simultaneously to estimate the overall probability of an outcome. While many machine learning algorithms ignore features that have weak effects, NB methods utilize all the available evidence. NB classification was conducted via R package "e1071". The parameter of positive double controlling Laplace smoothing is set as 1. The R code can be found in "S4 File".

*K-nearest neighbors* (KNNs) is known as an instance-based learning method, where the function of minimizing the mismatches is only approximated locally [64, 65]. KNN classification was conducted with R package "class" [66] with the number of neighbors considered parameter k = 20. The R code can be found in "S4 File".

### Evaluation metrics

Prediction accuracies as well as Sensitivity, Specificity, Precision, Decision Accuracy, and AUC were estimated for each of the 10-fold cross-validation outcomes for all 10 models.

#### Prediction accuracies

GBLUP and E-GBLUP genotypic values produced by RR and BGLR respectively were compared with the BLUP IDC values derived from the observed_IDC scores using r_pm_/h, the correlation coefficient [67] between BLUP genotypic values and either GBLUP or E-BLUP genotypic values divided by the square root of the reliability [67].

*Sensitivity* is the estimated frequency of lines that are truly tolerant to IDC [68]. Sensitivity is calculated as:

$$Sensitivity=\frac{\Sigma \;true\;positives\;(TP)}{\Sigma \;true\;positives+\Sigma \;false\;negatives}=\frac{\Sigma \;true\;tolerant\;lines}{\Sigma truetolerant\;lines+\Sigma \;false\;susceptible\;lines}$$

*Specificity* is the estimated frequency of lines that are correctly identified as susceptible to IDC [68]. Specificity is calculated as:

$$Specificity=\frac{\Sigma \;true\;negatives}{\Sigma \;true\;negatives+\Sigma \;false\;positives}=\frac{\Sigma \;true\;susceptible\;lines}{\Sigma \;true\;susceptible\;lines+\Sigma \;false\;tolerant\;lines}$$

*Precision* is used to evaluate the ability to identify truly tolerant IDC lines from among a group consisting of both truly tolerant lines and falsely identified tolerant lines. The higher precision (closer to 1), the lower risk of advancing lines susceptible to IDC.


$$Precision=\frac{\Sigma \;true\;positives\;(TP)}{\Sigma \;true\;positives+\Sigma \;false\;positive}=\frac{\Sigma \;true\;tolerant\;lines}{\Sigma true\;tolerant\;lines+\Sigma \;false\;tolerant\;lines}$$

*Decision accuracy* is the proportion of true positives and true negatives among all lines.


$$Accuracy=\frac{\Sigma \;true\;positive+\Sigma \;true\;negatives}{total}=\frac{\Sigma \;true\;tolerant\;lines+\Sigma \;true\;susceptible\;lines}{total\;lines}$$

*The receiver operating characteristic* (ROC) curve is generated by plotting the true positive rate (TPR) on the y-axis against the false positive rate (FPR) on the x-axis at various decision threshold settings. The ROC curve is plotted for all FPR values between 0 to 1. The area under the ROC curve (AUC) is used to estimate the model’s stability for making correct decisions about retaining and discarding lines. AUC values closer to 1 indicate that the model provides stable decision-making outcomes [69, 70]. The AUC was calculated with the "ROCR" R package [71]. The R code can be found in "S4 File".

### Comparisons of evaluation metrics among methods

Because these metrics have unknown distributions, the methods were ranked according to the metric values for each of the 10-fold cross-validation data sets and the Kruskal Wallis test was applied to the rankings. The Kruskal–Wallis test is a non-parametric method for testing whether the metrics’ median values are derived from the same distribution of outcomes based on Fisher’s least significant difference. The Kruskal–Wallis test was computed with R package "agricolae" where the significance level parameter was set to 0.05 (see "S6 File”).

In addition to comparing GP methods using each of the individual metrics, we combined rankings of decision accuracy and AUC for a combined assessment of the stability of correct decisions among all 10 methods using the Kruskal Wallis test. We also assessed patterns among the 10-fold cross validations for all five metrics using principal components analysis (PCA). PCA provided a method to identify correlated responses among the metrics as principal components for classification and visualization. The PCA was implemented with the "prcomp" function and parameter scale = True from R Package "stats". The resulting clusters based on the separation of PCAs were visualized with "pca3d". The R code can be found in, "S7 File."

## Results

The estimated reliability of IDC scores for the 1000 lines is 0.77. The prediction accuracy for the RR generated GBLUP genotypic values across 10 fold cross-validation sets is 0.73/0.88 = 0.83. The prediction accuracy for E-GBLUP genotypic values across 10 fold validation sets is 0.56/0.88 = 0.64.

From the perspectives of variety development, the more critical question is whether decisions based on genomic prediction models will be correct. If the LR method is used to build a model consisting of significant marker associations to predict the true decisions, then all of the decision metrics are about 0.5, indicating that results from the decisions using the LR method are not much better than tossing a coin (Table 1).

**Table 1:** Estimates of average ± Standard Deviation (SD) for five decision metrics derived from ten replicates of 10-fold cross-validation results created by 10 Genomic Prediction (GP) methods.

GP method	Sensitivity	Specificity	Precision	Decision accuracy	AUC
RR	0.86±0.03	0.83±0.02	0.89±0.02	0.85±0.01	0.76±0.05
BGLR	0.66±0.03	0.83±0.03	0.86±0.02	0.75±0.02	0.94±0.00
LR	0.51±0.06	0.5±0.06	0.51±0.07	0.50±0.05	0.51±0.04
PLR	0.86±0.04	0.88±0.05	0.89±0.05	0.87±0.03	0.97±0.02
ANN	0.91±0.03	0.90±0.03	0.90±0.04	0.90±0.02	0.99±0.02
RF	0.98±0.02	0.93±0.02	0.94±0.02	0.96±0.01	1.00±0.01
GBM	0.99±0.05	0.89±0.04	0.89±0.04	0.91±0.04	1.00±0.02
SVM	0.61±0.06	0.97±0.04	0.99±0.02	0.67±0.05	0.94±0.04
NB	0.94±0.03	0.74±0.06	0.78±0.06	0.84±0.04	0.91±0.04
KNN	0.79±0.06	0.83±0.06	0.83±0.05	0.81±0.03	0.47±0.06

The sensitivity of the LR, BGLR, and SVM methods is not very good, indicating that these methods have a tendency to incorrectly identify tolerant lines as susceptible to IDC. The most sensitive decisions were made using either the RF or GBM algorithmic modeling methods. While the sensitivity of the ANN method was not as good as for RF and GBM, it was better than any of the data modeling methods. The specificity of decisions had reasonably high values for all data modeling methods except LR. The SVM and RF methods provided the most specific decisions, indicating that they are best at identifying susceptible lines without incorrectly identifying susceptible lines as tolerant to IDC. The most precise methods included the SVM and RF methods. The SVM method will result in retaining IDC tolerant lines with almost no risk of incorrectly retaining false positive IDC lines. The RF and GBM methods had the best ability to correctly identify both susceptible and tolerant lines, i.e., they are the most accurate methods. According to the AUC values, RF, GBM and ANN are methods that produce the most stable decisions about classifying lines as either tolerant or susceptible. While the PLR and BGLR data modeling methods did not produce the most sensitive, specific, precise or accurate decisions, these two methods produced consistent decisions.

**Fig 2:**
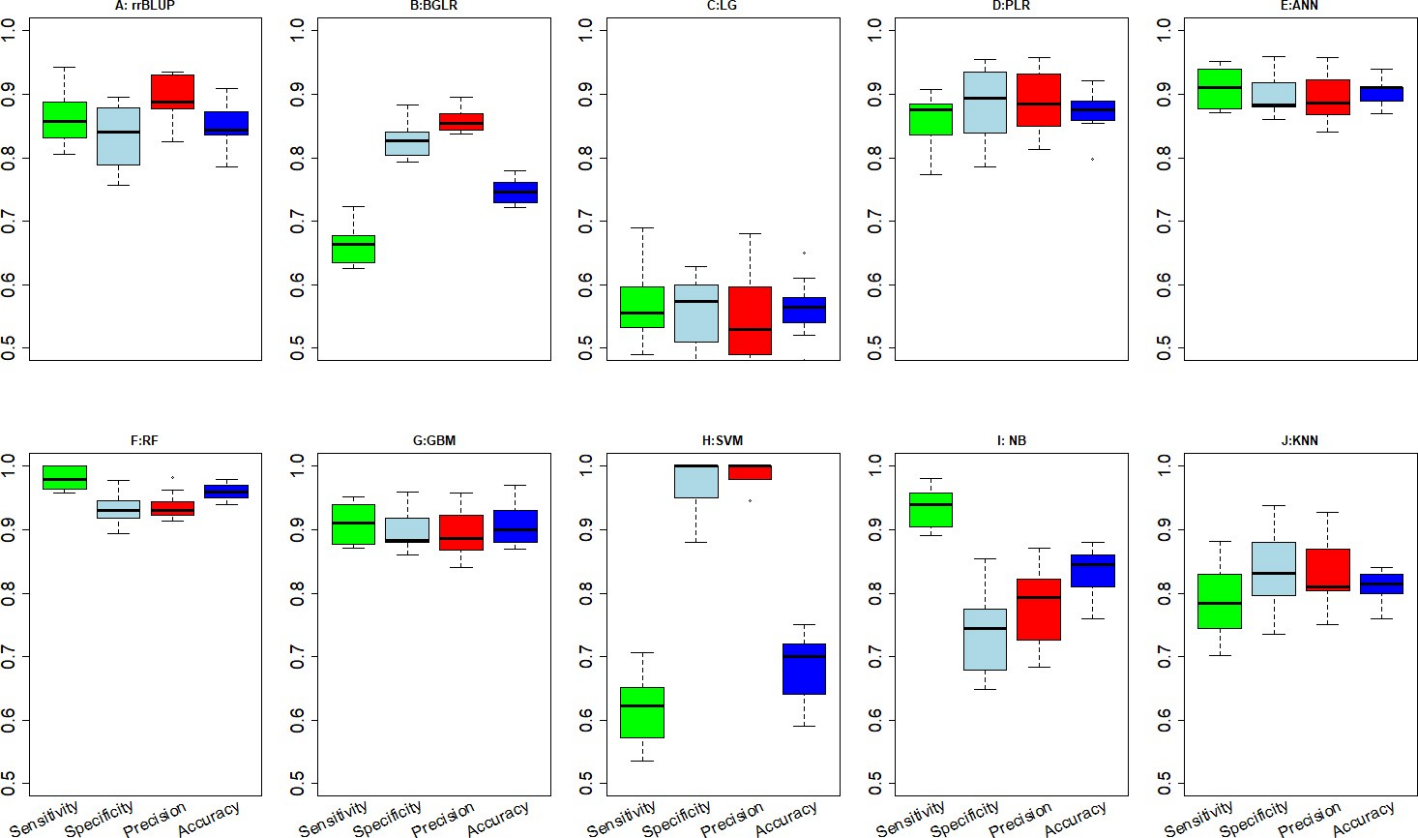
Boxplots from 10 fold cross validations of sensitivity, specificity, precision and decision accuracy for 10 GP methods. Y-axis values for these metrics range from 0.5 to 1.

**Fig 3:**
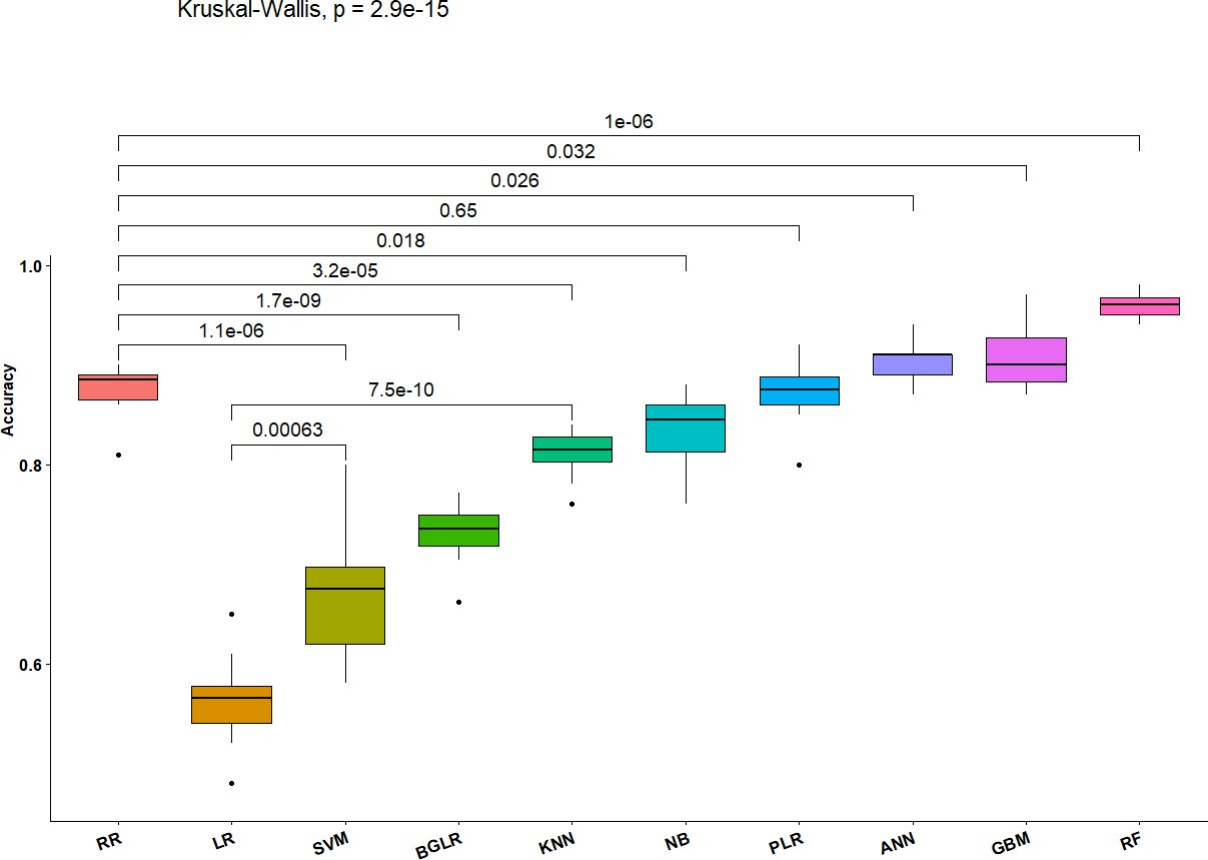
Boxplots of estimated decision accuracies from 10 replicates of 10-fold cross validations and significance of differences among ten GP methods based on Kruskal Wallace tests. The horizontal lines inside each box represent the medians. Black dots represent outliers, and the numbers above connecting lines at the top of the graphic are the p-values for pairwise comparisons, while the value for the line that transcends all methods at the top of the figure is the overall p-value from the Kruskal-Wallis test for multiple comparisons of ranked decision accuracy values.

Overall, the RF method resulted in among the best values for sensitivity, specificity, precision, accuracy, and AUC (Table 1, Figs 2 and 3), indicating that the decisions based on the RF method produced the fewest mistakes and most stable correct decisions. The GBM and ANN also consistently produced better metrics than any of the data modeling methods, although not significantly better than the PLR method.

The remaining three algorithmic models generally did better than the data modeling methods, but not uniformly. For example, the sensitivity of identifying truly tolerant lines among the lines using the SVM was 0.61, much less than RR and indicating that almost 40% of the truly tolerant lines would be mis-classified as susceptible using the SVM model. In contrast, the estimated specificity of the SVM indicates that only three to four percent of the truly susceptible lines would be incorrectly identified as tolerant (Table 1 and Fig 2). The differences between sensitivity and specificity suggest that the SVM is a useful method for cull IDC susceptible lines. In contrast to SVM, the NB model had a low specificity of 0.74 but a high sensitivity of 0.94 (Table 1 and Fig 2), indicating that the NB method can correctly predict the tolerant IDC lines better than it can predict susceptible lines. It may be appropriate for some breeding situations to conduct both SVM and NB genomic predictions. Unlike the SVM and NB models, the KNN model produced similar sensitivity and specificity values. Still, decisions using the KNN model are not as good as decisions from predictions using RR or PLR.

The boxplots associated with the Kruskal Wallace tests applied to Decision Accuracies (Fig 3) indicate that RF provided significantly more accurate decisions than RR. Further, the RF, ANN, PLR, KNN and RR methods are not only accurate but also stable. In contrast, the accuracy of SVM is not only low but also is highly variable.

**Fig 4:**
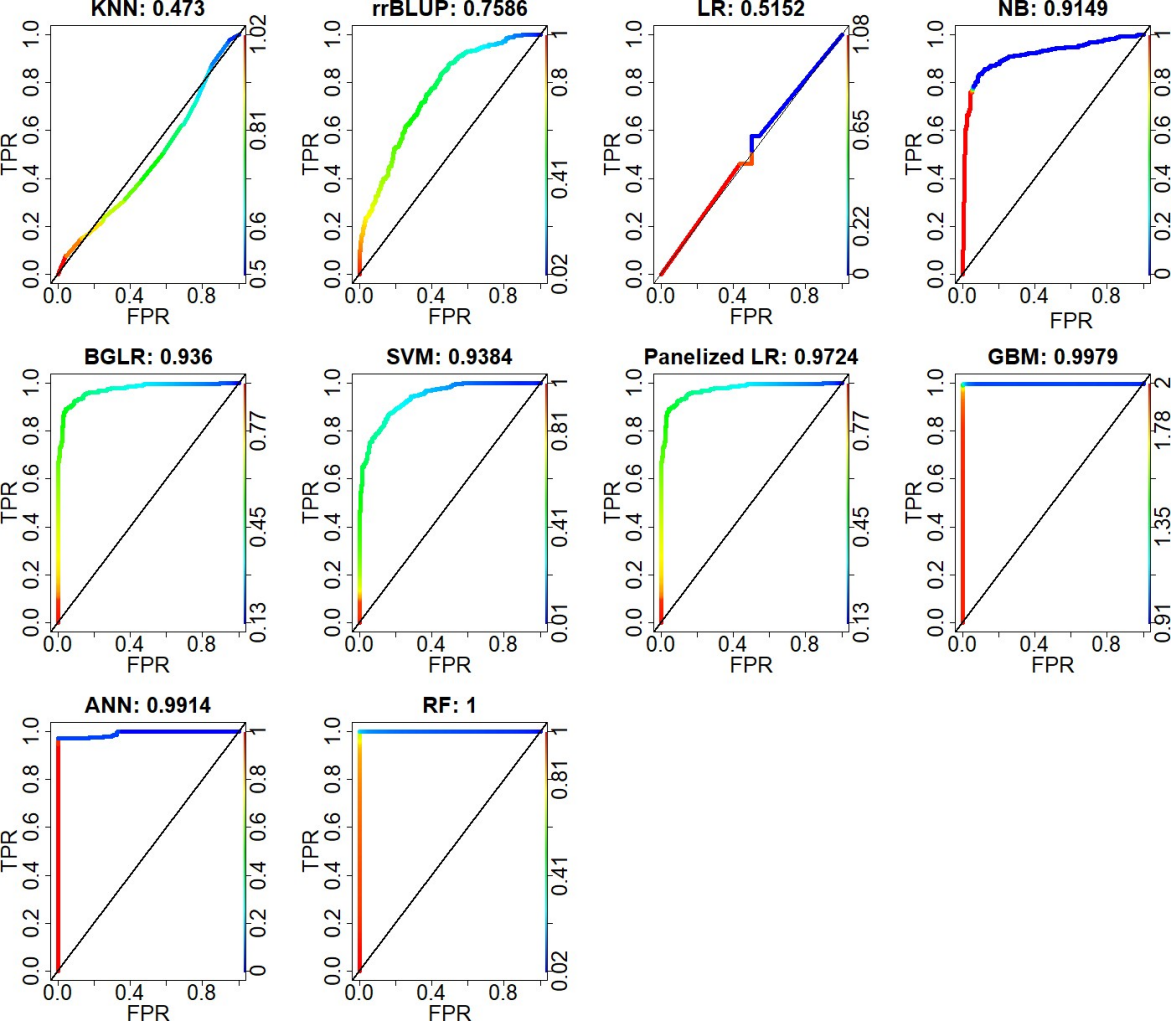
The area under the Receiver Operating Characteristic curve (AUC) from ten GP models. The X-axis is the false positive rate (FPR), the y-axis is the true positive rate (TPR). At the top border of each image, the number after the colon is the AUC value. A 45-degree diagonal line of each AUC is interpreted as random classifications with equal frequencies of TPR and FPR.

**Fig 5:**
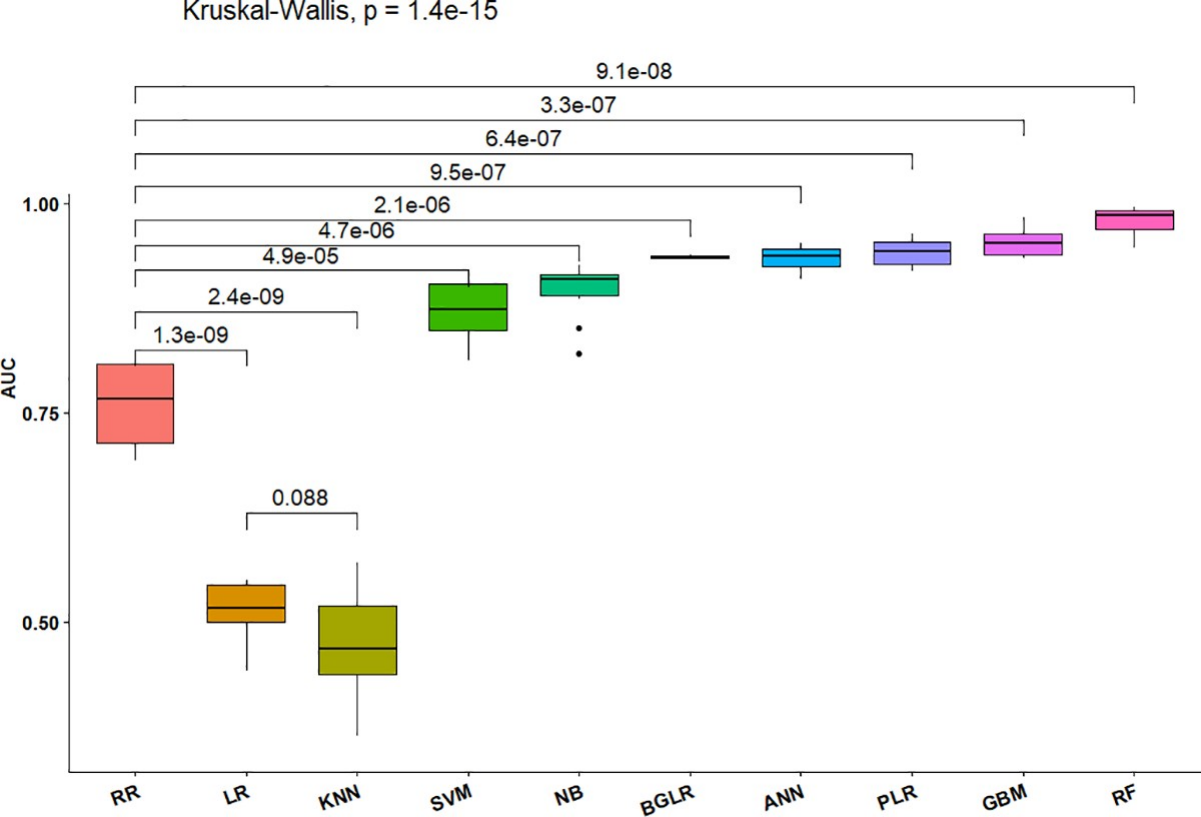
Summary of Kruskal-Wallis test comparisons of rankings of medians of AUC values generated by 10-fold cross-validation of decisions by 10 GP methods. The values above horizontal lines at the top of the graphic are the p-values associated with pairwise comparisons. The horizontal lines inside each box represent the medians. The black dots represent outliers.

The boxplots associated with the Kruskal Wallace tests applied to AUC affirm that KNN and LR have small AUC values, indicating both models are unable to provide stable decisions about which lines are tolerant which are susceptible to IDC (Figs 4 and 5). The AUC of SVM is lower than that of RF, which is consistent with an overall low accuracy but higher specificity provided by the SVM method. The KNN is the poorest algorithmic model with the lowest AUC value and highest standard deviation.

Results from the comparisons between pairs of AUC values indicate that LR and KNN are similar (p = 0.088; Fig 5) and provide the worst decision outcomes among the models. The SVM, NB, ANN, PLR, GBM, and RF have significantly greater (p-values < 0.001) AUC values than RR. Overall, the algorithmic models outperform the data models.

The combined rankings of decision accuracy and AUC indicate that the RF method provided the most stable and correct decisions among all 10 GP methods, while the LR method provided the least stable and greatest frequency of incorrect decisions (Table 2). The most stable and correct decisions using a data modeling approach were provided by the PLR method, although it was not as consistent at providing correct decisions as either the RF or GBM methods.

**Table 2:** Results of the Kruskal Wallis test applied to rankings of both decision accuracy and AUC values from 10-fold cross-validations produced by 10 GP methods.

Model Names	Kruskal-Wallis statistics	Fisher’s least significant difference (LSD) Groups(α = 0.05)
RF	183.675	a
GBM	148.7	b
ANN	135.325	bc
PLR	127.175	c
NB	99.5	d
rrBLUP	77.875	e
SVM	69.8	e
BGLR	47.3	f
KNN	39.15	f
LR	18.7	g

**Fig 6:**
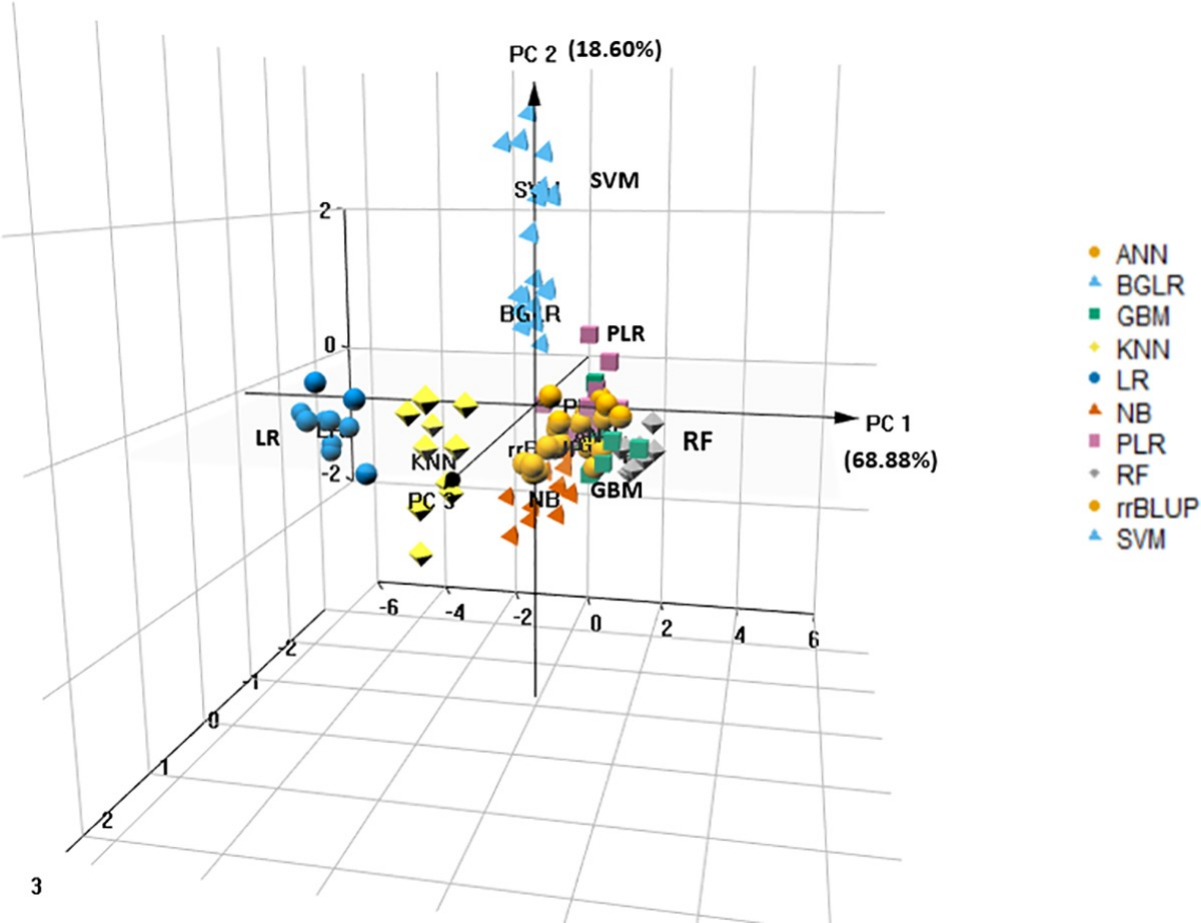
Visual representation of principal components of covariance among five decision metrics derived from ten replicates of 10-fold cross-validation results created by 10 GP methods. Principal components 1, 2, and 3 (PC1, PC2, and PC3) explain 0.68, 0.19, and 0.1 of the total variability.

Principle components 1 and 2 explain 68% and 19% of the total variation among the five sets of decision metrics obtained using 10 fold cross validations of GP using 10 methods. The three-dimensional plot of the eigenvectors for the first three principal components (Fig 6) shows the models’ tendencies to cluster based on similar patterns among the decision metrics. The SVM is differentiated from NB along with the second principal component, while LR and RF produced results that were the least related according to the first principal component.

## Discussion

Because conditions that enable IDC expression are ephemeral and difficult to predict [4], decisions about whether a line is tolerant to the adverse conditions often require field trials conducted at many environments (locations and years). Like disease expression traits, expression of IDC tolerance is opportunistic. It can only be evaluated visually in some parts of field trials that elicit a response in check varieties known to be tolerant or susceptible. Therefore, if GP models can accurately predict IDC, they will significantly reduce costs of variety development and genetic improvement [72].

Stroup [31] described how Fisher’s ANOVA and its implementation in user-friendly software forced plant breeders and agronomists to transform response variables into continuous variables. Indeed, IDC and many disease traits evolved from binary to ordinal scores and use of Normal approximations in an attempt to utilize ANOVA for purposes of assigning perceived statistical significance to decisions that had to be made in variety development and genetic improvement projects. Currently, most publicly supported soybean breeders use a range of ordinal values from 1 to 5, while commercial soybean breeders use a range of ordinal scores from 1 to 9. Stroup [31, 73] also described how GLMM provided plant breeders and agronomists with the freedom to utilize ordinal scores *per se*. GLMM methods for ordinal scores were developed with the explicit assumption that the visual score is an abstraction of some underlying unknown continuous variable (Fig 1). Further, he described how GLMM methods had been implemented in software packages, although he admitted that understanding and applying GLMM methods required unlearning the ANOVA paradigm and engaging with new steep learning curves.

Herein, we recognize that plant breeding is a decision-making discipline, so the metrics used to make decisions should reflect genetic improvement and variety development objectives. This led to a question about whether IDC ordinal scores are needed to predicting accurate binary decisions using established GP methods. To answer the question, we utilized high-quality experimental data (*i*^2^ = 0.78) and, more importantly, validated decisions about IDC tolerance and susceptibility. We then evaluated four data modeling and six algorithmic GP methods using metrics that quantify correct and incorrect decisions relative to the validated decisions.

Our results indicate that three algorithmic modeling GP methods produce better decision metrics than any data modeling GP methods, and the three remaining algorithmic modeling methods produce decision metrics that are equivalent to the best data modeling methods. Importantly for plant breeders, the black box algorithmic modeling methods do not require a steep learning curve associated with being able to specify distributional assumptions for the response variable and the random effect parameters representing the explanatory variables.

Our results are consistent with a previous report that algorithmic modeling outperforms Bayesian logistic ordinal regression in animal congenital diseases predictions. Two algorithmic machine learning models (RF and Boosting) provided better predictions than Bayesian logistic ordinal regression (Bayes A and Bayesian LASSO) for pig scrotal hernia disease [74]. Notably, the authors demonstrated that the machine learning algorithms were advantageous when a small number of simulated additive QTL were responsible for expressing the trait. Differences between the two approaches disappeared with a large number of simulated additive QTL. Given the results from simulations [74] and the observation that half of the algorithmic modeling methods were better than the data modeling methods that we evaluated, it is possible that tolerance to IDC in the evaluated germplasm is an oligogenic trait.

As a practical matter, it difficult to recommend a single algorithmic modeling method. The RF model provides the best trade-off among all decision metrics (Tables 1 and 2), but combinations of algorithmic modeling methods are needed to maximize all decision metrics. Using a combination of RF and SVM, it would be possible to minimize undesirable decisions to less than 4% across all decision metrics.

We only investigated one of several possible LMMs, one of many possible multinomial threshold models, and one of many possible PLR models [33]. Thus, it may be possible to find data modeling methods that will produce better selection decisions than the algorithmic modeling methods. However, it is not our intention to provide a comprehensive assessment of all possible data modeling methods. Rather, our intention is to redirect attention from data analysis methods to metrics that accurately reflect the breeding objectives.

## Conclusions

As Stroup [31] pointed out, we should not adapt our experimental methods to available analysis tools. Our intent is to extend this line of thinking a little further: We should first ask the right questions then find or develop analytics to address these. A comparison of GP models applied to ordinal soybean IDC scores revealed that algorithmic modeling approaches provided better decisions than data modeling approaches, consistent with Breiman’s conclusions about the two statistical modeling cultures [26]. Among the ten GP models, the SVM demonstrated the best ability to discard IDC susceptible lines, while the RF method demonstrated the ability to accurately and consistently select IDC tolerant lines.

## Supporting information

S1 File (R)

S2 File (R)

S3 File (R)

S4 File (RMD)

S5 File (R)

S6 File (R)

S7 File (R)

S8 File (CSV)

S9 File (CSV)

S10 File (CSV)
